# Comparison of glucose concentration measured in samples collected with different anticoagulants and analyzed using 2 glucose quantification methods

**DOI:** 10.3168/jdsc.2024-0730

**Published:** 2025-04-01

**Authors:** Anay D. Ravelo, Megan Ruch, Isaac J. Salfer, Luciano S. Caixeta

**Affiliations:** 1 Department of Veterinary Population Medicine, University of Minnesota, Saint Paul, MN 55108; 2 Department of Animal Science, University of Minnesota, Saint Paul, MN 55108

## Abstract

Sodium fluoride (NaF), K_2_-EDTA, and lithium heparin are common anticoagulants used during collection of bovine blood for glucose concentration. Whereas NaF is considered the best anticoagulant for preservation of glucose, other anticoagulants are used because of their ability to simultaneously preserve other metabolites frequently measured in dairy science experiments. To simplify sample collection and reduce the need for multiple tubes, it may be possible to use tubes with anticoagulants other than NaF, provided they yield comparable glucose concentrations. Additionally, blood glucose concentrations are commonly analyzed using a peroxidase and glucose oxidase (PGO) colorimetric assay with a standard plate reader, but the use of a semi-automated chemistry analyzer with a hexokinase (HK) enzymatic reaction method is becoming more common in dairy research because it is also available and easy to use. Thus, the objective of this study was to determine the influence of the use of NaF, dipotassium EDTA (K_2_-EDTA), or lithium heparin (Li-Hep) as anticoagulants on glucose quantification, using both the PGO and HK enzymatic reaction methods. Blood samples from 20 fresh cows (7 ± 3 DIM) were collected into NaF, K_2_-EDTA, or Li-Hep plasma tubes on a commercial dairy farm (n = 60 samples). All samples were analyzed using both the PGO method, performed on a 96-well plate with a colorimetric microplate reader, and the HK method, conducted on a semi-automated chemistry analyzer. It was observed that in both methods, samples collected into NaF tubes had less glucose quantified compared with samples collected into K_2_-EDTA and Li-Hep tubes. Additionally, it was observed that in samples quantified with the HK method, the glucose concentrations were lower than when those samples were quantified with the PGO method. Thus, the K_2_-EDTA and Li-Hep anticoagulant could be used for glucose quantification to avoid cumbersome collection of several tubes, but results must be reviewed with caution as concentrations would be higher compared with those quantified with NaF. This observation is also conserved when quantifying glucose using the PGO and HK methods.

## Highlights

• Sodium fluoride yielded lower glucose concentrations compared with Li-Hep.
• Sodium fluoride yielded lower glucose concentrations compared with K_2_-EDTA.
• Enzymatic methods used for glucose measurement yielded different concentrations.

## Graphical Abstract

**Figure:**
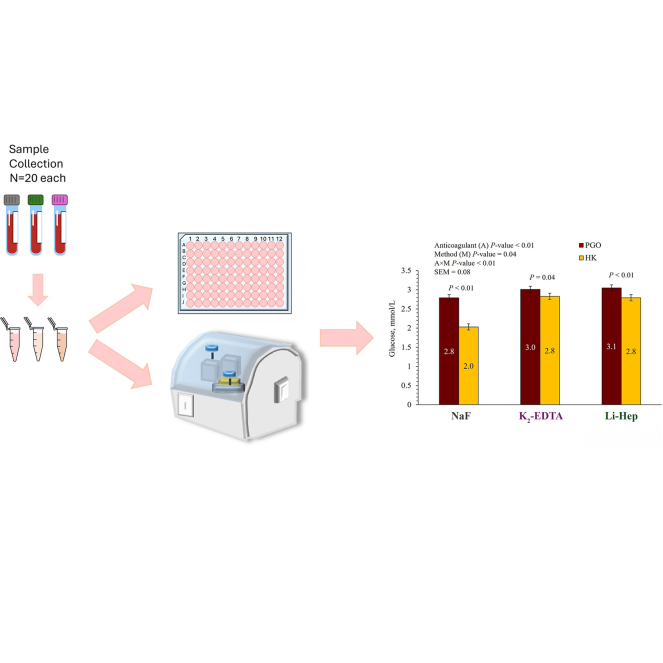
**Summary:** Blood samples were collected from 20 cows into 3 tubes with different anticoagulants, including sodium fluoride (NaF), dipotassium ethylenediaminotetraacetate (K_2_-EDTA), and lithium heparin (Li-Hep) tubes. Two different enzymatic reactions, peroxidase and glucose oxidase (PGO) and hexokinase (HK), were used for quantification of glucose concentration in the plasma samples collected with the different anticoagulants. Across both methods, samples collected in NaF yielded lower glucose concentrations compared with K_2_-EDTA and Li-Hep. Additionally, the samples quantified with HK yielded lower glucose concentrations than the same samples quantified with PGO.

In dairy cattle research, glucose concentrations found in blood or plasma are often of interest because glucose is an important metabolic fuel (Horst et al., 2021). Glucose serves as the precursor for production of lactose, the sugar found in milk (McCarthy et al., 2020), and acts as the osmotic regulator of milk synthesis. Additionally, during the transition stage, glucose availability can influence immune response and milk production (Horst et al., 2021), and affects insulin secretion, which is one of the regulators of the homeorhetic responses to the start of lactation. Knowing glucose concentration in the days around calving may also be important for predicting or monitoring responses to treatments administered to dairy cows (Hubner et al., 2022). Therefore, it is crucial to have precise and reproducible measures of plasma glucose concentration.

There are different anticoagulants used for preservation of metabolite concentrations in plasma and serum. Sodium fluoride (NaF) is an anticoagulant that is conventionally preferred for the preservation of glucose concentration because NaF prevents glycolysis (Bayot and Tadi, 2023). Other anticoagulants often used for collection and plasma separation of dairy cow blood include dipotassium EDTA (K_2_-EDTA) and lithium heparin (**Li-Hep**; Caixeta et al., 2017; Gammariello et al., 2024). When sampling many cows in a farm or research setting, collecting samples into multiple tubes containing different anticoagulants could be cumbersome and time consuming if multiple metabolites are being analyzed from the same cow. Thus, if a singular anticoagulant could be used for the quantification of glucose and other metabolites, it would save time during the collection and processing, allowing for faster storage of samples.

Additionally, different enzymatic methods could be used for the quantification of glucose. These include a peroxidase and glucose oxidase (**PGO**) enzyme preparation or a hexokinase (**HK**) enzymatic reaction method (Fiedorova et al., 2022). Protocols using these methods are described in our companion article (Ravelo et al., 2025), describing discrepancies when comparing glucose concentrations quantified using both methods. Both colorimetric assays have been used in dairy cow research (Mann et al., 2016; Martin et al., 2021) because they are accessible and relatively easy to use.

Therefore, the objective of this study was to determine the impacts of anticoagulant present within the collection tube on the apparent glucose concentration in blood collected from dairy cattle using 2 different enzymatic methods of analysis. It was hypothesized that apparent glucose concentration would differ based on anticoagulant present in the collection tube; specifically, it would be greater with NaF, and based on method of analysis.

The sample size required to address this research question was calculated based on findings from our companion article (Ravelo et al., 2025) investigating the influence of different analytical methods (PGO vs. HK assays) for glucose quantification. The mean difference observed between the methods was 0.48 mmol/L with a SD of 0.68 mmol/L. Thus, for an effect size of 0.70 for Cohen's d, which was obtained by dividing 0.48 mmol/L by 0.68 mmol/L. Using a power of 0.80 and an α of 0.05 for a paired *t*-test, it was determined that 18 samples would be needed to detect the expected difference in glucose concentration in our sample size calculation. Samples were collected from cows enrolled in 2 ongoing observational studies within our research group. The University of Minnesota Institutional Animal Care and Use Committee approved procedures for animal care and handling (protocol number: 2211-40523A).

To compare 3 different anticoagulants, including NaF (BD Vacutainer Fluoride Tubes, BD Vacutainer), K_2_-EDTA (BD Vacutainer EDTA Tubes), and lithium heparin (Vacuette Tube 3 LH Lithium Heparin; Greiner Bio-One), one blood sample per anticoagulant was concurrently collected in consistent order from the coccygeal vessel in 20 fresh cows (7 ± 3 DIM; 85% were multiparous) on a commercial dairy farm. The NaF sample was collected first, followed by the K_2_-EDTA and then the Li-Hep sample. All samples from all cows at a given farm were collected within a 30-min window and then transported to the laboratory on ice and processed 3 ± 1 h after collection. Samples were centrifuged at 2,000 × *g* for 15 min at 4°C and the plasma was separated into 1-mL aliquots, stored in polypropylene microcentrifuge tubes (111562LK, Globe Scientific Inc.) and frozen at −20°C.

Samples were thawed at room temperature 2 wk after sample collection and simultaneously analyzed using 2 different methods: PGO enzyme preparation (Sigma Aldrich, St. Louis, MO; Shaker and Swift, 2023) analyzed on a 96-well plate (Greiner Bio-One, Frickenhausen, Germany) using a conventional plate reader (Eom, BioTek Instruments), at 450 n*M*, and a HK reagent (C-124-07, Catachem Inc.) with a small-scale chemistry analyzer (Chemwell-T Chemistry Analyzer, Awareness Technology Inc.)

For the 96-well microplate analysis, 5 µL of sample or standard was pipetted into each well. Then, 250 µL of the PGO solution was added using a multichannel pipette. The plate was shaken for 10 s and then was allowed to sit in the dark for 45 min before the absorbance was read at a wavelength of 450 nm using the plate reader (Eom, BioTek Instruments). For the automated chemistry analyzer (Chemwell-T Chemistry Analyzer, Awareness Technology Inc.), the samples and HK reagent were loaded into the analyzer. Through the automated pipetting, 300 µL of reagent was transferred into cuvettes. The reagent absorbance was read as a baseline at 350 nm, before 10 µL of sample was added to each corresponding cuvette well and agitated. Then the mixture was allowed to sit for 5 min before the absorbance was read again at 350 nm.

The standard curve used for both analyses included the glucose concentrations of 0, 1.1, 2.2, 3.3, 4.4, and 5.5 mmol/L. Pool samples were created using 3 randomly selected samples per anticoagulant that were observed to have visibly low hemolysis. The intra- and inter-assay CV were determined among the different anticoagulants measured within the different methods of analysis. The pool samples that were used in all analyses were observed to have consistent absorbances as the intra- and inter-assay CV were low and below 5% (Table 1).

**Table 1:** The intra- (Intra) and inter-assay (Inter) CV (%) for pool samples from the same samples that were collected using different anticoagulants, which included sodium fluoride (NaF), dipotassium EDTA (K_2_-EDTA), and heparin plasma (Li-Hep), and the concentrations were quantified using 2 common methods of analysis: a peroxidase and glucose oxidase (PGO) enzymatic reaction method and a hexokinase (HK) enzymatic reaction

Pool sample	Method of analysis			
	PGO		HK	
	Intra	Inter	Intra	Inter
NaF	0.93	4.90	1.29	4.32
K_2_-EDTA	1.67	3.43	1.45	3.25
Li-Hep	3.57	4.89	1.88	3.55

All statistical analyses were performed using R studio (https://www.r-project.org/). Bland–Altman (**BA**) plot, matched *t*-tests, and Pearson correlations were used to compare glucose concentrations of samples collected with K_2_-EDTA and Li-Hep to those collected with NaF within each enzymatic method of quantification. The *ggplot()* function from the *ggplot2* package (Wickham, 2016) was used to create the BA plots and the *ggscatter()* function from the *ggpubr* package (Kassambara, 2016) was used to illustrate the Pearson correlations. These were used to obtain the data presented in Table 2. Additionally, a mixed linear model was performed using the *lmer()* function of the *lme4* package (Bates et al., 2015), which included the fixed effect of anticoagulant, and random effect of cow was used to compare anticoagulants within enzymatic methods of analysis. Finally, a linear mixed model with the fixed effect of anticoagulant, method, their interaction, and the random effect of cow was used to consider the quantification of glucose by anticoagulant and method of analysis. For all BA plots and mixed models, the studentized residuals that exceeded ±3 SD away from the mean were removed, and a Tukey adjustment was used for pairwise comparisons in mixed models. The outliers that were removed were one K_2_-EDTA measured concentration that was low from the HK method and one Li-Hep measured concentration from the PGO method that was high; however, the removal of these points did not change the results.

**Table 2:** The comparison of glucose concentrations across different anticoagulants was measured by 2 methods of analysis including a peroxidase and glucose oxidase (PGO) enzymatic reaction method and a hexokinase (HK) enzymatic reaction^1^

Item	Mean^2^	BA (95% CI)^3^	*t*-test (95% CI)^4^	*t P*-value^5^	r^6^	*P*-value^7^	SEM^8^	*P*-value^9^
PGO								
NaF	2.79^b^	—	—	—	—	—	0.10	0.01
K_2_-EDTA	3.01^a^	−0.22 (−0.91, 0.47)	(−0.38, −0.06)	0.01	0.66	<0.01		
Li-Hep	3.05^a^	−0.31 (−1.28, 0.66)	(−0.54, −0.08)	0.01	0.53	0.02		
HK								
NaF	2.03^b^	—	—	—	—	—	0.08	<0.01
K_2_-EDTA	2.83^a^	−0.73 (−1.62, 0.15)	(−0.94, −0.52)	<0.01	0.55	0.01		
Li-Hep	2.79^a^	−0.75 (−1.46, −0.04)	(−0.92, −0.58)	<0.01	0.55	0.01		

a,b Different letters represent pairwise comparisons that were different based on Tukey adjustment for multiple comparisons within analysis methods (*P* < 0.05).

1 Samples were collected from 20 cows. Each cow had 3 tubes collected, which included sodium fluoride (NaF), dipotassium EDTA (K_2_-EDTA), and heparin plasma (Li-Hep).

2 The mean glucose concentration (mmol/L) for the anticoagulant.

3 The Bland–Altman (BA) plot difference of the means and the CI detected for the comparison NaF and the corresponding anticoagulant.

4 The matched *t*-test 95% CI; the *t*-tests all had the same mean difference concentrations as the BA plots.

5 *P-*value for the matched *t*-tests.

6 Pearson correlation coefficient for each anticoagulant with NaF.

7 *P-*value for the Pearson correlations.

8 The SEM for the mixed model comparing the mean concentrations, which included the fixed effect of anticoagulant used and the random variable of cow.

9 The *P*-value for the mixed model comparing the mean concentrations, which included the fixed effect of anticoagulant used and the random variable of cow.

The results from the analysis of the different anticoagulants with the 2 methods are presented in Table 2. It was observed that within both methods the glucose concentration quantified with NaF was lower than that quantified with K_2_-EDTA and Li-Hep. When comparing the glucose concentration determined using the PGO method, the concentration in NaF samples were 0.22 mmol/L lower than in K_2_-EDTA samples (*P* = 0.01). Similarly, when comparing NaF to Li-Hep, the glucose concentration in NaF was 0.31 mmol/L lower (*P* = 0.01). Using the HK analysis, the glucose concentration quantified in NaF samples were 0.73 mmol/L lower compared with K_2_-EDTA (*P* < 0.01) and 0.75 mmol/L lower compared with Li-Hep (*P* < 0.01). The agreement between NaF and the other anticoagulants within method was moderate (Table 2).

For all anticoagulants considered, the PGO enzyme method yielded greater concentrations of glucose compared with the HK method (Figure 1). The greatest discrepancy between the PGO and HK methods occurred from samples collected using NaF tubes. The difference in the glucose concentration when comparing both methods with the NaF anticoagulant was of 0.75 mmol/L (*P* < 0.01). The differences with the other 2 anticoagulants were smaller. The smallest difference was observed when K_2_-EDTA was used as the anticoagulant with a difference of 0.17 mmol/L (*P* = 0.04) between both methods, and for Li-Hep the difference was 0.26 mmol/L (*P* < 0.01).

**Figure 1:**
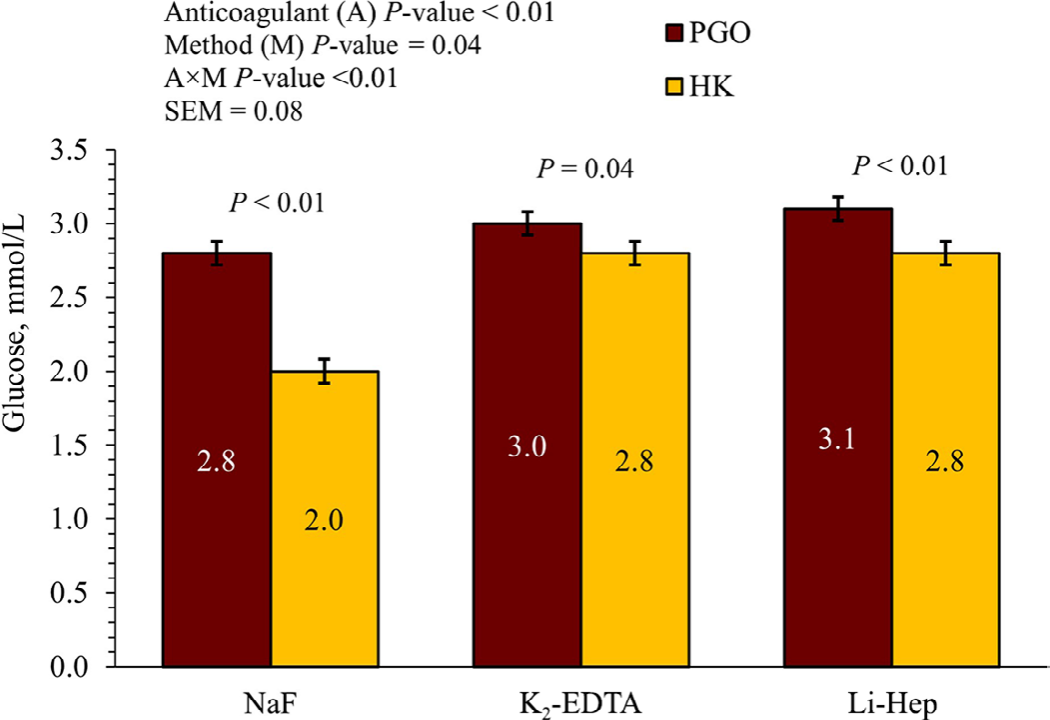
Comparison of the glucose concentrations that were quantified from samples collected from the same cow (n = 20) with different anticoagulants measured using 2 common enzymatic methods for glucose quantification including a peroxidase and glucose oxidase (PGO) enzymatic reaction method and a hexokinase (HK) enzymatic reaction. Each cow had 3 tubes collected, which included a sodium fluoride (NaF), a dipotassium EDTA (K_2_-EDTA), and a heparin plasma (Li-Hep) tube. The error bars are the SE.

Given the lower concentrations quantified by the HK assay with NaF, the authors speculate that the ability of HK to provide accurate glucose concentrations may be hindered by hemolysis in samples. Hemolysis was observed but not recorded after processing in samples collected with NaF anticoagulant. Hemolysis was not observed for the other plasma samples after processing. It has been previously demonstrated that with NaF anticoagulant, the cell membrane can be disrupted and hemolysis of red blood cells is increased due to the fluoride present (Bowen and Remaley, 2014). The hemolysis may affect the colorimetric assay specifically with HK because the color change is visually transparent. That may explain the greater difference in the concentrations quantified for glucose preserved with NaF across the PGO and HK methods of analysis as compared with K_2_-EDTA and Li-Hep. The PGO method seemed to be more accurate in its ability to read the hemolyzed sample absorbances because the enzymatic conversion creates a color change that is more of a copper color.

Overall, it was observed that in both methods of analyses, the NaF anticoagulant, which is generally considered to be most appropriate for collection of samples for the determination of glucose, was the one with the lowest glucose concentrations as compared with samples collected using other anticoagulants (K_2_-EDTA and Li-Hep). However, in the recommendations for laboratory analysis in diagnosis of diabetes mellitus in humans, it is not recommended that NaF alone be used as the enolase inhibitor to prevent glycolysis (Sacks et al., 2011). Instead, tubes with a citrate buffer that would rapidly inhibit glycolysis are now recommended. Additionally, in another study, it was observed that adding NaF to K_2_-EDTA tubes was not necessary for blood samples centrifuged within 2 h of collection (Drackley, 2023), as it did not provide any additional benefit for glucose preservation compared with only the K_2_-EDTA tube. In that report, it was also observed that K_2_-EDTA yielded lower concentrations than the heparin plasma. A further decrease in concentration was observed when fluoride was added to the K_2_-EDTA tube, supporting the idea that NaF does not necessarily lead to greater measured glucose concentrations. In the current study, however, the concentrations of glucose from samples collected with K_2_-EDTA and Li-Hep were comparable within both methods of analysis. The results suggest that greater glucose concentrations are quantified from the same samples with the use of K_2_-EDTA and Li-Hep as anticoagulants compared with when NaF is used, agreeing with Drackley (2023). Thus, contrary to our hypothesis, K_2_-EDTA and Li-Hep did not yield lower glucose concentrations as compared with NaF.

A limitation of the study was that samples were not processed with 30 min of collection; however, although not measured they were collected within a 30-min window and thus the gap between sampling and processing should have been similar for all the samples. Another limitation is that the samples were not analyzed in a reference laboratory, preventing a direct comparison of glucose measurement with those from a clinical pathology laboratory. Reference laboratories would likely be using the hexokinase method for glucose quantification. However, this would not affect the results because the tests in the current study are not being used for diagnostic testing. Finally, another limitation of the study is the fact that hemolysis in NaF samples was only visually assessed and was not evaluated on a scale.

Overall, the NaF samples had lower glucose concentrations quantified in both methods of analyses compared with the K_2_-EDTA and Li-Hep. Thus, K_2_-EDTA and Li-Hep tubes could be used for the quantification of glucose in cow blood, even when samples are processed within 3 h of collection. When quantifying glucose with the HK method, hemolysis of the sample appears to influence the readability of absorbances more than with the PGO method. Thus, comparisons of glucose concentration quantified from NaF in PGO and HK methods are not comparable and will yield different conclusions. However, measurements can still be used and compared as long as there is consistency in the anticoagulant and analytical method used. Future studies should consider the influence of hemolysis of plasma samples on colorimetric assays.
